# Epicardial Adipose Tissue Thickness and Preserved Ejection Fraction Heart Failure

**DOI:** 10.1007/s11906-024-01302-7

**Published:** 2024-04-20

**Authors:** Aneesh Dhore-Patil, Daniela Urina-Jassir, Rohan Samson, Thierry H. Le Jemtel, Suzanne Oparil

**Affiliations:** 1 Division of Cardiovascular Imaging, Weill Cornell Medical College, Houston Methodist DeBakey Heart & Vascular Center, 6505 Fanin St., Houston, TX 77030 USA; 2 Section of Cardiology, John W. Deming Department of Medicine, Tulane Avenue, SL-48, New Orleans, LA 70112 USA; 3 Advanced Heart Failure Therapies Program, University of Louisville Health-Heart Hospital, 201Abraham Flexner Way, Suite 1001, Louisville, KY 40202 USA; 4 Vascular Biology and Hypertension Program, Division of Cardiovascular Disease, Department of Medicine, University of Alabama at Birmingham, Birmingham, AL 35294 USA

**Keywords:** Heart failure with preserved ejection fraction, Obesity, Epicardial fat, Visceral adiposity, Elevated body mass index

## Abstract

**Purpose of the Review:**

Preserved ejection fraction heart failure and obesity frequently coexist. Whether obesity plays a consistent role in the pathogenesis of preserved ejection fraction heart failure is unclear. Accumulation of visceral adiposity underlies the pathogenic aftermaths of obesity. However, visceral adiposity imaging is assessed by computed tomography or magnetic resonance and thus not routinely available. In contrast, epicardial adiposity thickness is assessed by echocardiography and thus routinely available. We review the rationale for assessing epicardial adiposity thickness in patients with preserved ejection fraction heart failure and elevated body mass index.

**Recent Findings:**

Body mass index correlates poorly with visceral, and epicardial adiposity. Visceral and epicardial adiposity enlarges as preserved ejection fraction heart failure progresses. Epicardial adiposity may hasten the progression of coronary artery disease and impairs left ventricular sub-endocardial perfusion and diastolic function.

**Summary:**

Epicardial adiposity thickness may help monitor the therapeutic response in patients with preserved ejection failure heart failure and elevated body mass index.

## Introduction

Besides prevention of water and salt retention, the pharmacologic treatment of preserved ejection heart failure (HFpEF) focuses on common metabolic and cardiovascular comorbidities such as hypertension (HT), type 2 diabetes (T2D), and arterial stiffening [1]. However, obesity and associated conditions play an increasing role in the development and progression of HFpEF [2]. Obesity contributes to the development and progression of HFpEF through accumulation of visceral and epicardial adipose tissue (VAT, EAT) that promotes low-grade systemic inflammation and adipokine dysregulation [3, 4]. Thus, imaging VAT or EAT may be helpful when assessing the response to therapy in patients with HFpEF and obesity.

Magnetic resonance (MR) and computed tomography (CT) imaging allow direct and accurate measurement of VAT and EAT mass [5, 6]. As both imaging modalities are expensive, with CT exposing subjects to ionizing radiation and MRI being unpractical in severe obesity, VAT and EAT volumes are not readily obtained in clinical practice. Although two-dimensional (2D) echocardiography is an unreliable imaging modality for measurement of EAT volume, it reliably determines EAT thickness, a useful estimate of EAT in clinical settings [7, 8].

We review the development, characteristics, and clinical implications of VAT and EAT in the syndrome of HFpEF and advocate routine determination of EAT thickness in the management of patients with HFpEF.

## Visceral Adipose Tissue

The amount of VAT is typically measured as the area of omental and mesenteric adipose tissue (AT) on a single abdominal cross-sectional slice by MR or CT at 5–6 cm above the L4-L5 disc [9]. Alternatively, the amount of VAT may be measured in 3 abdominal cross-sectional slices at L2-L3, L3-L4, and L4-L5 intervertebral spaces and average values derived from the 3 slices [10].

In response to excess intake of nutrients, lipids are stored as triglycerides (TGs) in the subcutaneous (SC) adipose tissue (SAT) surrounding the flanks, hips, buttocks, and thighs to mold the “pear shape” pattern of obesity [3, 11, 12]. When nutrient intake continues to exceed energy expenditure, the SC adipocytes become tenfold larger than in the basal state and SAT undergoes considerable accumulation [13]. When SAT reaches its maximum capacity for TG storage, visceral adipocytes start accumulating TG in the omental and mesenteric adipose depots followed by non-neuronal organs (liver, skin, vasculature, kidney, ovaries, adrenal glands, skeletal muscle, and heart) [13–16]. Regional specific differences among AT depots exist in pre-adipocyte proliferation, apoptosis, and differentiation capacity [11].

Visceral adiposity increases the risk of cardiovascular disease independent of total body adiposity [17]. Adipocyte lineages in the VAT and EAT originate from the visceral mesothelium that lines the internal organs [16]. Accumulation of VAT reflects the inability of SAT to act as a metabolic sink and to protect organs from toxic metabolites such as fatty acyl-coAs, diacylglycerides, and ceramides [3, 18, 19]. However, in turn, VAT accumulation fails to prevent storage of lipids in the liver, heart, pancreas, and skeletal muscle although it may delay it [20]. As obesity steadily progresses, reduced functional activity lowers energy expenditure thereby widening the caloric intake-energy expenditure gap and heightening the lipid storage burden [21]. Further, visceral adipocytes have a restrained capacity for lipid storage owing to a limited increase in size and a potential for hyperplasia [13]. Although visceral adipocytes do not enlarge as much as SC adipocytes, VAT accumulation triggers a stronger inflammatory compared to SAT and results in extensive visceral adipocyte necrosis and fibrosis [15, 16]. Specific adaptive AT responses to accumulation are important determinants of AT health and systemic metabolic homeostasis. Obesity-induced alterations in AT metabolism, extracellular matrix formation, immune system function, and inflammation regulate metabolic function in several organs. Differences in these factors likely contribute to heterogeneity in metabolic health in obesity [15].

## Epicardial Adipose Tissue

**Fig. 1:**
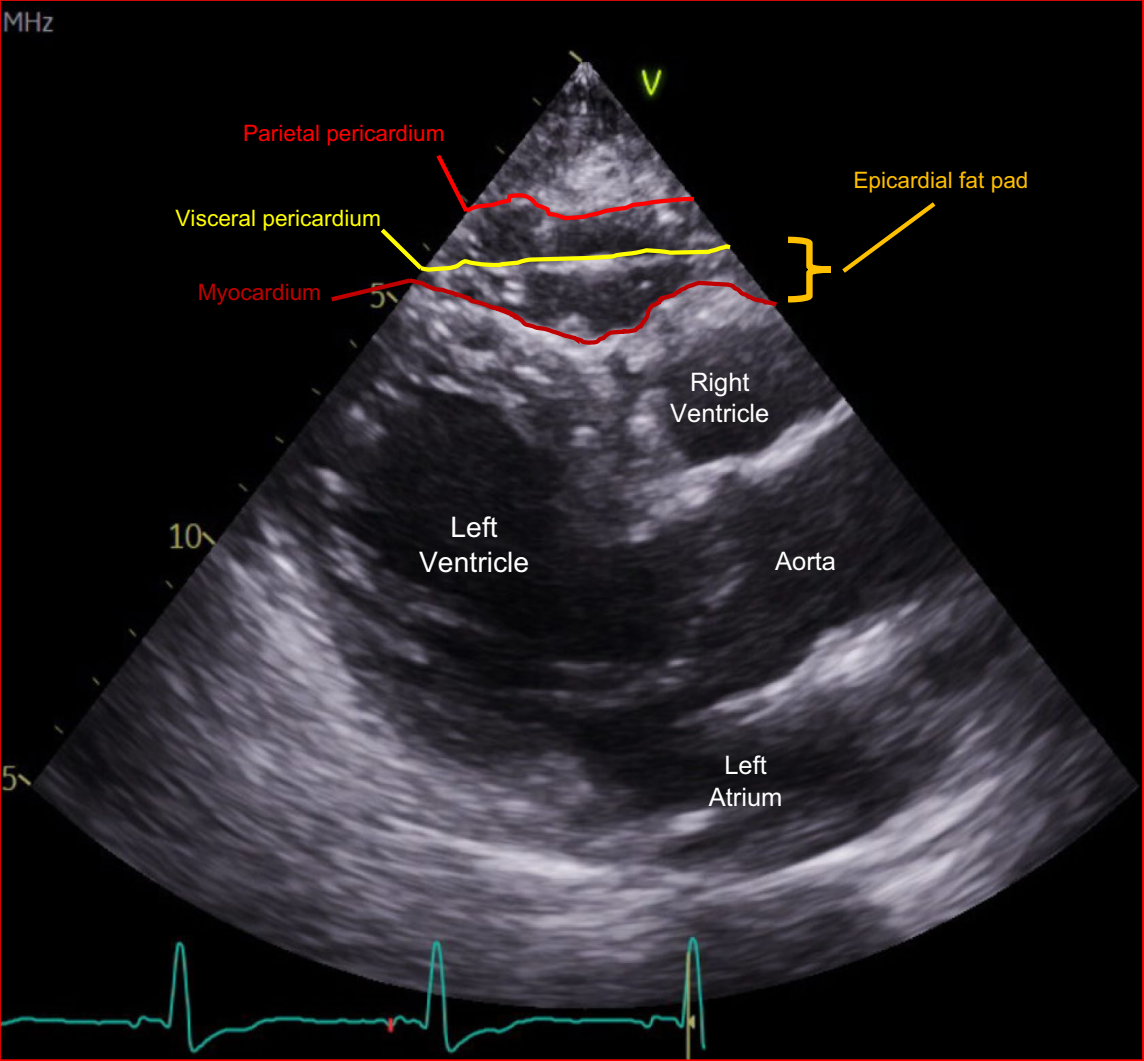
Parasternal long axis window from transthoracic echocardiogram, depicting a layer of epicardial fat between the myocardium and epicardium

Lying between the myocardium and visceral pericardium, EAT covers most of the right ventricle (RV) and part the left ventricle (LV) with an EAT-myocardium ratio of 0.48 for the RV and 0.15 for the LV [22] (Fig. 1). Epicardial AT surrounds large coronary arteries, and their branches as EAT occupies atrial-ventricular and interventricular grooves [23]. The thickness of healthy EAT ranges from 5 to 7 mm over the RV free wall and from 10 to 14 mm in atrial-ventricular and interventricular grooves [24]. The amount of EAT correlates weakly with body mass index (BMI) [25]. Although age, waist circumference, ethnicity, and myocardial hypertrophy are independent determinants of EAT, one unfrequently adjusts EAT thickness [26]. Both EAT and VAT originate from the splanchnopleuric mesoderm with EAT being vascularized by the coronary artery network [27].

The contiguity of adipocytes and stromal vascular fraction (resident inflammatory cells plus lymphocytes [CD3^+^], macrophages [CD68^+^], and mast cells) to coronary arteries underlies the vascular effects of EAT [28, 29••, 30]. Accumulation of EAT in the left atrioventricular groove has a strong association with coronary atheromatous plaques while excessive VAT contributes to the development of the metabolic syndrome [31]. Further, accumulation of EAT and VAT was associated with coronary atheromatous plaques in 174 patients with suspected coronary artery disease (CAD) with only EAT predicting the presence of coronary calcifications [32]. In 45 patients with CAD, EAT volume indexed to body surface area was greater around functionally significant coronary stenosis (mean fractional flow reserve [FFR] of 0.74) than around non-significant stenosis (mean FFR of 0.89) 0.34 vs 0.27 ml/m^2^, *p* = 0.045 [33]. Yu et al. corroborated the association between EAT volume and hemodynamically significant CAD in 164 patients [34].

Local expression of chemokine monocyte chemotactic protein [MCP]-1, interleukin [IL]-1β, IL-6, and tumor necrosis factor [TNF]-α RNA and protein is greater in EAT than in SAT from patients with critical CAD [28]. Heightened EAT inflammation may foster the development of coronary artery lesions. Alternatively, atherosclerosis-induced inflammation may propagate to contiguous AT [35, 36]. Nevertheless, the plasma concentration of circulating inflammatory cytokines does not reflect the degree of EAT inflammation [28]. Secretion of adiponectin, an anti-inflammatory adipokine, is 40% lower in patients with than in patients without CAD [17]. Dysregulated levels of novel adipokines and pro-inflammatory cells in EAT compared to VAT underlie the strong contribution of EAT to the pathogenesis of CAD [37]. Catecholamine-stimulated synthesis and release is greater in EAT than in other AT depots [17]. Last, protein content is greater and glucose oxidation capacity is lower in EAT than in VAT [17].

Epicardial AT expression of TNF-α is greater in patients with than in patients without non-calcified coronary plaques and is independent from coronary calcium score and clinical status [38]. Enhanced AT expression of TNF-α and increased vascular expression of endogenous endothelin (ET)-1 and ET receptor A (ET_A_) contribute to imbalance of the endothelin [ET]-1/nitric oxide [NO] system by impairing tonic NO release [39]. Adiponectin gene expression and thus concentration decreases significantly in epicardial adipocytes as coronary artery atherosclerosis progresses [40]. Epicardial AT expansion is inversely related to perfusion of LV sub-endocardial layers and LV global longitudinal strain in patients with CAD; sub-endocardial layer perfusion and LV strain are directly related [41]. Analysis of macrophage polarization markers reveals increased low-grade inflammation in EAT biopsies from patients with CAD [42]. Epicardial AT inflammation and neo-angiogenesis correlate with the presence of non-calcified plaques and coronary calcifications in patients with and without obstructive CAD [38]. The contribution of EAT to the progression of coronary atherosclerosis is well recognized [43, 44]. However, the usefulness of EAT attenuation for risk stratification and prediction of major acute cardiac events remains controversial in patients with CAD [45–47].

In patients with non-atherosclerotic vascular disease, AT surrounding arteries may release mediators that regulate vascular smooth muscle cell proliferation, matrix degradation, and neo-revascularization [48]. Release of cytokines, free fatty acids, exosomes carrying protein, lipids, ribonucleic acids (RNAs), and miRNAs from EAT to vascular smooth muscle and endothelial cells through the coronary arterial wall vasa vasorum may underlie local inflammation and coronary microvascular dysfunction [29••]. The interaction between perivascular adipose tissue (PVAT) and obesity sheds light on the impact of EAT on the coronary vasculature. In lean subjects, PVAT attenuates the vascular responsiveness to phenylephrine, angiotensin II, and ET-1 by releasing adipocyte- or perivascular-derived relaxing factors [49]. In patients with obesity, PVAT does not exert the anti-contractile effect to phenylephrine, angiotensin II, and ET-1 that PVAT exerts in lean patients [50]. The obesity triad of hypoxia, local inflammation, and oxidative stress upregulates pro-inflammatory cytokine expression in PVAT and downregulates that of adiponectin and anti-inflammatory cytokines thereby counteracting the anti-contractile effect of PVAT in lean subjects [49]. Associated with myocardial hypertrophy and capillary rarefaction, obesity causes local hypoxia that leads to fibrosis and necrosis of cardiomyocytes.

The volume of EAT by CT correlates inversely with myocardial blood flow reserve (MFR) estimated by rubidium-82 (^82^Rb) positron emission tomography in patients with normal myocardial perfusion imaging and no coronary artery calcifications [51]. Patients with increased EAT volume and no obstructive CAD display reduced global LV longitudinal strain (GLS) and normal global LV circumferential and radial strain [52]. Selective LV sub-endocardial layer dysfunction, as evidenced by a decrease in LV GLS, argues against myocardial fat infiltration as the cause of LV GLS decrease. Increased EAT volume may reduce MFR, thereby decreasing sub-endocardial layer perfusion and LV GLS [53]. Hence, impaired LV sub-endocardial perfusion mediates LV diastolic dysfunction in women with obesity, HFpEF, and no CAD [54]. Independent of general measures of adiposity, increased EAT thickness correlated with coronary microvascular function in 399 elderly patients [55]. Myocardial and hepatic TG contents were measured by proton magnetic resonance spectroscopy (^1^H-MRS), and LV function VAT volume and EAT area were measured by MR imaging in 75 non-diabetic subjects with hepatic steatosis [56]. Hepatic TG content was low in 26 subjects, moderate in 24, and high in the remaining 25 subjects. Subjects with high and moderate hepatic TG content had 2–threefold higher myocardial TG content than those with low TG hepatic content. Hepatic TG content and VAT were inversely related to LV diastolic function. In contrast, myocardial TG content was unrelated to LV diastolic function. Thus, myocardial lipotoxicity may not contribute to LV diastolic dysfunction in patients with increased VAT and EAT.

## Epicardial Adipose Tissue and Preserved Ejection Fraction Heart Failure

The thickness of EAT over the RV in parasternal long- and short-axis echocardiographic views indexed to body surface area predicted incident HFpEF over a mean follow-up of 4.3 years in 379 patients with CAD and no overt HF [57]. The predictive value of EAT was independent of age, BMI, and sex. However, patients, who developed HFpEF, had presumably latent HFpEF as they were receiving more loop diuretics and renin–angiotensin–aldosterone inhibitors at enrollment in the study than patients who did not develop HFpEF were receiving [57]. Patients with HFpEF, elevated BMI, and increased EAT thickness display higher LV eccentricity index and lower peak functional capacity than their counterparts with normal EAT thickness [58]. Similarly, HFpEF patients with increased EAT thickness and BMI > 30 kg/m^2^ have a lower peak oxygen uptake after adjustment for pulmonary vascular resistance and BMI than patients with BMI < 30 kg/m^2^ and normal EAT thickness [59]. Increased EAT thickness closely correlates with arterial stiffness in HFpEF patients [60]. The mechanisms that link EAT to increased arterial stiffness remain poorly understood. The volume of EAT by MRI predicted a composite clinical outcome of all-cause mortality and first HF hospitalizations over a median follow up of 2 years in 105 patients with mid-range and preserved ejection heart failure whose BMI averaged 30 kg/m^2^ [8]. The predictive value of EAT was independent of BMI, age, sex, HF severity, and several comorbidities. Systemic HT was the most prevalent comorbidity; it affected 80% of patients whose mean systolic blood pressure was 140 mmHg at baseline despite treatment. As expected, 52% of patients with high EAT volume had CAD compared to 23% in patients with low EAT volume. Last, VAT was not measured and thus not entered as covariate in the multivariable Cox proportional hazard regression models.

## Epicardial Versus Visceral Adipose Tissue in Preserved Ejection Fraction Heart Failure

The majority of cardiometabolic studies that assess EAT volume/thickness reports a consistent relationship between EAT and clinical outcomes [61–64]. However, cardiometabolic studies that conjointly assess the amount of VAT and cardiac AT assert that VAT mass is the overwhelming pathogenic factor in obesity except for an association between ectopic cardiac AT and coronary artery calcification or atrial fibrillation [18, 19, 26, 65].

The relative impact of AT distribution (epicardial versus visceral) on LV diastolic function may depend on the amount of VAT [66••]. Accumulation of EAT contributes to LV diastolic dysfunction in patients with low VAT mass and does not contribute to LV diastolic dysfunction in patients with high VAT mass [66••]. Both VAT and EAT contribute to LV diastolic dysfunction in patients with recent myocardial infarction (MI) [67]. However, the association between VAT and LV diastolic dysfunction is much stronger than the association between total adiposity and LV diastolic dysfunction in patients with recent MI [67]. Last, only VAT accumulation correlates with sub-clinical LV diastolic dysfunction in patients with end-stage renal disease on peritoneal dialysis [68].

## Epicardial Adipose Tissue Thickness as an Endpoint in Clinical Trials

A composite of cardiovascular mortality and HF hospitalization is the prevailing primary endpoint in HF therapeutic trials. However, the incidence of cardiovascular mortality is relatively low in patients with HFpEF. Cardiovascular mortality was 8.9% over 35 months in the placebo arm of the sacubitril/valsartan HFpEF trial [69]. Non-cardiac comorbidities are highly prevalent in patients with HFpEF [70, 71]. Patients with HFpEF are as likely to be hospitalized for decompensated HFpEF as they are for worsening of non-cardiac comorbidities [72]. The incidence of HF hospitalizations was only 14.6% over 39 months in the placebo arm of the spironolactone HFpEF trial [73]. Further, HFpEF patients with high BMI are at increased risk of non-HF hospitalizations due to obesity-related comorbidities like cellulitis, deep vein thrombosis, gastrointestinal-esophageal reflux disease, or respiratory illness [74].

A randomized, placebo-controlled trial recently reported that 52 weeks with 2.4 mg of semaglutide, a glucagon-like peptide 1 receptor agonist (GLP1-RA), significantly improved HF-related symptoms and physical limitations in patients with HFpEF and a median BMI of 37.2 kg/m^2^ [75]. Kansas City Cardiomyopathy Clinical Questionnaire clinical summary score (KCCQ-CSS) and 6-min walk distance increased as C-reactive protein (CRP) level decreased in patients randomized to semaglutide. Whether the improvement in KCCQ-CSS and 6-min walk distance resulted from improvement(s) in obesity-related cardiac/peripheral disturbances or from a -13.3% loss body weight is unclear [75]. Semaglutide reduced CRP levels by 44, 39, and 48% in the Semaglutide Treatment Effect in People with Obesity (STEP) 1, 2 and 3 trials, and caloric reduction-induced weight loss increased peak oxygen uptake by 1.8 ml/min/kg in patients with HFpEF [76, 77]. Inclusion of epicardial adiposity as an endpoint in obesity therapeutic trials may help uncover how anti-obesity medication or procedures benefit elevated BMI patients with HFpEF besides a straight weight loss [22, 78–80].

In summary, visceral adipose tissue plays a larger role in the development and progression of LV diastolic dysfunction than epicardial adipose tissue although the latter may have a local impact on the coronary circulation and thereby on LV diastolic function. However, assessment of visceral adipose tissue mass requires CT or MR and thus is not routinely available in patients with HFpEF. In contrast, 2D echocardiography, which provides a reliable estimate of epicardial adipose tissue thickness, is routinely available in patients with HFpEF. Differentiating a healthy from an unhealthy metabolic status is challenging in patients with HFpEF and obesity as HT, T2D, and dyslipidemia are shared comorbidities [81]. Increased epicardial adipose tissue thickness points to a pathogenic role of obesity in patients with HFpEF and elevated BMI. Increased epicardial adipose tissue thickness signals the need to add anti-obesity medications or procedures to standard HFpEF therapy.
